# Matrix-comparative genomic hybridization from multicenter formalin-fixed paraffin-embedded colorectal cancer tissue blocks

**DOI:** 10.1186/1471-2407-7-58

**Published:** 2007-04-02

**Authors:** Heiko Fensterer, Bernhard Radlwimmer, Jörn Sträter, Malte Buchholz, Daniela E Aust, Catherine Julié, François Radvanyi, Bernard Nordlinger, Claudio Belluco, Eric Van Cutsem, Claus-Henning Köhne, Hans A Kestler, Carsten Schwaenen, Michelle Nessling, Manfred P Lutz, Peter Lichter, Thomas M Gress

**Affiliations:** 1 Department of Internal Medicine I, University of Ulm, Robert-Koch-Strasse 8, 89081 Ulm, Germany; 2 Department of Pathology, University of Ulm, Robert-Koch-Strasse 8, 89081 Ulm, Germany; 3 Division of Molecular Genetics, German Cancer Research Center, Im Neuenheimer Feld 280, 69120 Heidelberg, Germany; 4 Department of Pathology, University of Technology, Fetscherstraße 74, 01307 Dresden, Germany; 5 Department of Internal Medicine, Caritasklinik, Rheinstraße 2, 66113 Saarbrücken, Germany; 6 Department of Pathology Hôpital Ambroise Paré, Boulogne, France; 7 UMR144 CNRS – Institut Curie, 26 rue d'Ulm, 75248 Paris Cedex 05, France; 8 CRO – IRCCS, National Cancer Institute, Via Pedemontana Occidentale, 12, 33081 Avianov(PN), Italy; 9 Digestive Oncology Unit, University Hospital Gasthuisberg, 3000 Leuven, Belgium; 10 Department of Hematology, University of Ulm, Robert-Koch-Strasse 8, 89081 Ulm, Germany; 11 Department of Neural Information Processing, University of Ulm, 89069 Ulm, Germany; 12 Division of Gastroenterology and Endocrinology, Department of Internal Medicine, Philipps University, Baldingerstrasse, 35043 Marburg, Germany; 13 Division of Oncology and Hematology, Klinikum Oldenburg, Dr.-Eden-Str.10, 26133 Oldenburg, Germany

## Abstract

**Background:**

The identification of genomic signatures of colorectal cancer for risk stratification requires the study of large series of cancer patients with an extensive clinical follow-up. Multicentric clinical studies represent an ideal source of well documented archived material for this type of analyses.

**Methods:**

To verify if this material is technically suitable to perform matrix-CGH, we performed a pilot study using macrodissected 29 formalin-fixed, paraffin-embedded tissue samples collected within the framework of the EORTC-GI/PETACC-2 trial for colorectal cancer. The scientific aim was to identify prognostic genomic signatures differentiating locally restricted (UICC stages II-III) from systemically advanced (UICC stage IV) colorectal tumours.

**Results:**

The majority of archived tissue samples collected in the different centers was suitable to perform matrix-CGH. 5/7 advanced tumours displayed 13q-gain and 18q-loss. In locally restricted tumours, only 6/12 tumours showed a gain on 13q and 7/12 tumours showed a loss on 18q. Interphase-FISH and high-resolution array-mapping of the gain on 13q confirmed the validity of the array-data and narrowed the chromosomal interval containing potential oncogenes.

**Conclusion:**

Archival, paraffin-embedded tissue samples collected in multicentric clinical trials are suitable for matrix-CGH analyses and allow the identification of prognostic signatures and aberrations harbouring potential new oncogenes.

## Background

Genomic copy number changes are frequently found in different types of cancer and are believed to contribute to their development and progression through inactivation of tumour suppressor genes, activation of oncogenes, or more subtle through gene dosage changes. Comparative genomic hybridization (CGH) [1] was developed to allow genome-wide screening for such copy number changes. Conventional CGH has a limited resolution and can detect losses of 10 Mb or greater [2]. High-level amplifications achieve a maximum resolution of 3 Mb [3]. The resolution of CGH has been improved by replacing the metaphase chromosomes, which have traditionally served as hybridization targets, with mapped and sequenced genomic DNA clones (bacterial artificial chromosomes, P1-derived artificial chromosome or cosmids) arrayed onto glass slides which was named "matrix-CGH" [4] or "array-CGH" [5].

Although genomic DNA arrays are considered powerful research tools, their potential to meet specific needs in clinical diagnostics has been debated. Initially, matrix-CGH was restricted to cell lines or investigated inherited diseases, both characterized by a genetically homogeneous cell population [4,5]. More recently, it was also used in research studies addressing issues of tumour classification and correlation of gene dosage with gene expression studies [6].

Genomic arrays allow the identification of genomic copy number alterations that may be suitable for individualized diagnostic, prognostic and therapeutic decision making. This is of particular importance since personalized treatments for cancer patients based on genomic alterations are becoming available. Ideally this type of analysis is performed using DNA from tumour samples freshly collected and snap frozen in liquid nitrogen. However, this type of tissue is not always available in clinical routine. Furthermore, in order to identify prognostic alterations, the analysis of large numbers of well documented tumour samples with ample and accurate clinical follow-up data is required. This information is usually available in controlled multicentric therapeutic studies. Many large-scale multicentric therapeutic studies such as those from the EORTC ("European Organisation for Research and Treatment of Cancer") already mandate the collection and storage of formalin-fixed paraffin-embedded tumour samples in their study protocols [7], and archival paraffin tissue-blocks can be collected for most patients that participated in past large multicentric trials which would have the additional benefit of an extended follow up. Unfortunately, current protocols for matrix-CGH require microgramm quantities of high quality DNA [8]. Such material can usually not be obtained from the formalin-fixed, paraffin embedded specimens making these valuable resources unavailable for the search of prognostic genomic alterations. Different protocols to overcome this problem were published [9,10]. Recently, DeVries and coworkers [10] compared hybridizations with DNA extracted from formalin-fixed paraffin-embedded breast cancer tissue samples after manual microdissection and with DNA derived from the same fresh frozen tumour. In this study, reproducible results from archived formalin-fixed paraffin-embbeded tissue samples were obtained using as little as 50 ng input DNA. However, the conditions available in these studies only partly reflect the situation found in routine clinical work or in large multicentric studies. Their tissue blocks were collected by an expert center and were all derived from one institution, thus ensuring comparable and high-quality tissue processing. In most clinical trials, tissue blocks will be collected by a large number of centers, many of them without any expertise in the processing of samples for molecular analyses and using different protocols for fixation and embedding.

So far it is not known, whether DNA of acceptable quality for molecular analyses such as matrix-CGH can be obtained from this type of material in a multicentric setting. In this report, we present a pilot project initiated by the translational research group of the EORTC-GI group addressing this particular question. To test the technical suitability of this type of material, we performed matrix-CGH analysis of macrodissected colorectal tumours collected in the framework of the EORTC-GI PETACC-2 trial. In addition to testing the technical feasibility of this approach, the scientific aim of this pilot project was to identify prognostic genomic signatures differentiating locally restricted (UICC stages II-III) from systemically advanced (UICC stage IV) disease.

## Methods

### Patients and tumour specimens

29 paraffin-embedded colorectal tumour samples in different stages (10 × UICC II, 11 × UICC III, 8 × UICC IV) were collected in the framework of the PETACC-2 study. 23 of these samples were from patients which participated in the trial, and 6 additional samples were generated from archival material collected by PETACC-2-participants. The samples were provided by the EORTC-GI group. From one tumour (no. 29) we did also obtain snap frozen tissue. The retrospective use of tissue blocks from these patients for translational research was approved by the ethics committee of the "Ärztekammer Niedersachsen" (Berliner Allee 20, 30175 Hannover, Germany, reference number Grae/128/2002).

Tissue samples were collected at four institutions (University of Padua, Italy, Hôpital Ambroise Paré, Boulogne, Paris, France, Department of Pathology, University of Technology, Dresden and Department of Pathology University Hospital of Ulm, Germany). All specimens were fixed in formalin and paraffin-embedded using the standard protocols active in the participating centers. Fixation times were not controlled for and did probably vary. For diagnostic purposes, paraffin sections of about 3 μm were performed and stained H&E. For isolation of tumour-specific DNA, paraffin sections of about 50 μm containing only tumour tissue after macrodissection of adjacent normal tissue were collected.

### DNA extraction and MSI-testing

DNA extraction of the paraffin embedded tumours was done using a standard protocol with proteinase K digestion and phenol/chloroform extraction [11], which yielded between 3 and 15 μg DNA from one section. A detailed protocol is provided as part of the supplementary material (additional file 1). In tumour no. 29 DNA was also extracted from the snap frozen tissue using a standard protocol [12]. As a control we used DNA from peripheral leucocytes of healthy volunteers as previously described [13]. Tumour microsatellite instability status was determined using the BAT26 microsatellite marker following a standard protocol [14]. Tumours were defined as having high-frequency microsatellite instability (MSI-H) if change of length mutations were detected in BAT26 when compared with DNA from colon cancer cell lines with known MSI-status. Because of the quasi monomorphic nature of the BAT26 polyA tract (size variation is uncommon between germline alleles), this marker can be used to screen initially for MSI without matching normal DNA [15].

### Array production

Two different types of microarrays were used. DNA from all tumours was hybridized to a chip previously described by members of our group [13] containing 644 DNA targets. For the fine mapping of a selected gain on chromosome 13q in one tumour we used an additional chip containing 6400 DNA targets. These comprise 3200 clones with a genome-wide resolution of 1 MB (Sanger-Institute, Cambridge, Great Britain) and 2800 additional clones of regions showing frequent genomic alterations in cancer or that are known to harbor oncogenes and tumour suppressor genes. Lists of the clones included in the arrays together with their genomic localization are provided as supplementary material (additional files 2 and 3). Clone preparation and spotting was done as previously described by our group [6,13].

### Array hybridization

Tumour and control DNA (each 250 ng) were labelled with Cy3 and Cy5-conjugated dCTP by random priming. Each DNA sample was labeled separately with both fluorophores and used for independent array-hybridizations ("Colour-switch"). Labelled tumour and control DNA was hybridized to the chip. Detailed protocols are available as supplementary material on the journals webpage (additional file 4).

### Data Acquisition and Evaluation

Images of fluorescence signals were acquired by a dual laser scanner (GenePix 4000 A, Axon Instruments, Foster City, CA). Assessment of fluorescence signal intensities was done using GENEPIX PRO 4.0 imaging software. To identify imbalanced genomic sequences we used a specialized algorithm previously developed and validated by our group [9]. Array experiments were declined for further analysis if the R value of the Gauss fit did not reach 0.96 (see [9]). In contrast to this protocol, we made no grading of imbalances: all imbalances were described as gains or losses, simple gains were not differentiated from amplifications.

### Fluorescence in-situ hybridization (FISH)

Fluorescence in-situ hybridization (FISH) was performed as previously described for metaphases [16] and interphases [17]. Two-color FISH to 2 μm-sections of the formalin-fixed samples was performed with the Rhodamin-labelled BAC-clones RP11-89P22 (27.7 MB) and RP11-8C15 (21 MB) with corresponding FITC-labelled BAC-clone RP11-9F13 (40.7) as a control. To verify their location on 13q all these probes were hybridized with leucocytes of healthy volunteers before interphase FISH. The gain was shown in relation to the control by visual analysis of the hybridization signals. Criteria for gene amplification were: tight clusters of signals in multiple cells or at least three times more test probe signals than control signals per cell in > 10% of the tumour cells (a minimum of 100 cells were counted).

## Results

### Quality of DNA extracted from formalin-fixed paraffin-embedded colorectal cancer samples

**Figure 1:**
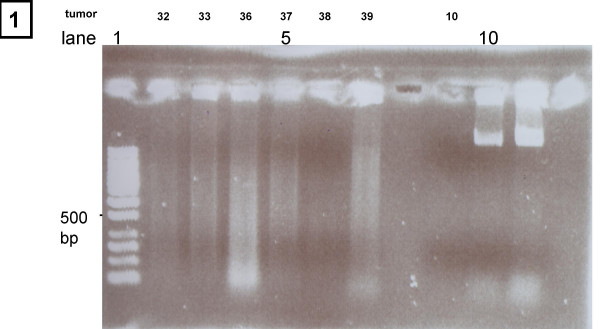
Comparison of different DNA samples after extraction: Lane 1: DNA-ladder, 2–7 and 9: DNA from paraffin-embedded colorectal cancer samples, lane 8: no DNA loaded, lane 10+11: DNA from cultured cells.

**Figure 2:**
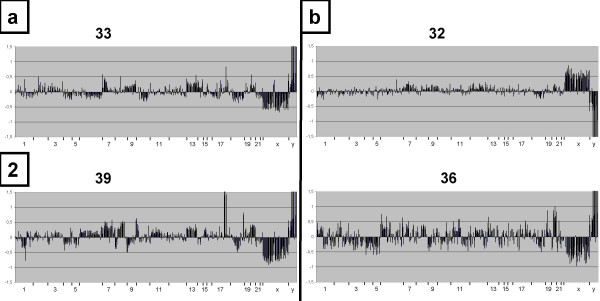
Comparison of the different matrix-CGH-profiles from DNA showing moderate (figure 2a) and higher degree of degradation (figure 2b). Log_2 _ratios for the different BAC clones (y-axis) are displayed according to their chromosomal position (x-axis).

A total of 3–15 μg DNA per section suitable for array-hybridizations could be extracted from 22 tissue blocks, no DNA or only highly degraded DNA was extractable from 7 tissue blocks. The clinical characteristics of these patients are shown in table 1. The quality of the extracted DNA was checked on agarose gels. Figure 1 shows a representative gel with lanes showing no DNA at all (lanes 6 and 9), DNA with a high degree of degradation (lanes 2 and 4 showing the bulk of the DNA between 100 and 1500 bp) and with a moderate degree of degradation (lanes 3, 5 and 7 bulk of DNA between 400 and 3000 bp). Best array hybridization results were obtained with DNA showing a moderate degradation as in lanes 3 (no. 33) and 7 (no. 39) (Figure 2a), however, interpretable results could as well be obtained with DNA showing a higher degree of degradation such as the DNA in lanes 2 (no. 32) and 4 (no. 36) (Figure 2b). For 3 of the samples, the array quality was too poor to include them in the further analysis (the R value of the Gauss fit did not reach 0.96). There was no correlation of DNA or array quality with the age of the tumor block.

**Table 1:** Patient characteristics of the 29 patients in which DNA extraction was performed ("+" = good quality, "-" = bad quality, n.d. = not done, F = female, M = male, n.k. = not known, n.a. = not available)

Code	DNA quality	Interpretable profile	Age	Sex	Site	G	T	N	M	UICC Stage	Date of sample preparation	MSI status
1	+	no	72	F	left colon	2	2	x	1	4	06/01	n.d.
2	+	no	67	M	left colon	2	3	1	1	4	03/00	neg.
3	+	yes	63	M	right colon	2	3	2	0	3	05/98	n.d.
4	+	yes	75	F	right colon	2	2	2	1	4	10/00	n.d.
5	+	yes	78	M	left colon	2	3	1	0	3	03/95	neg.
6	+	yes	80	M	rectum	2	3	0	0	2	03./99	n.d.
7	+	yes	75	M	left colon	2	2	1	0	3	02/98	n.d.
8	-	n.d.	65	F	left colon	2	2	2	0	3	04/99	n.d.
9	+	yes	60	M	rectum	2	3	1	0	3	01/99	neg.
10	-	n.d.	65	M	right colon	2	3	0	0	2	02/00	n.d.
11	+	yes	43	M	rectum	2	3	1	0	3	12/98	n.d.
13	-	n.d.	64	M	coecum	3	3	1	0	3	10/97	n.d.
15	+	yes	66	F	rectum	2	3	0	0	2	01/92	n.d.
17	+	no	58	F	left colon	2	2	1	0	3	07/94	n.d.
18	+	yes	51	M	left colon	2	3	0	0	2	11/97	neg.
19	+	yes	57	M	left colon	2	3	1	0	3	07/92	neg.
21	neg.	n.d.	53	F	rectum	2	3	0	0	2	10/97	n.d.
22	neg.	n.d.	40	F	left colon	2	4	1	0	3	05/98	n.d.
23	+	yes	64	F	left colon	1	3	0	0	2	11/92	neg.
25	+	yes	62	F	right colon	2	3	1	1	4	10/01	neg.
26	neg.	n.d.	67	F	left colon	3	3	0	0	3	12/99	n.d.
27	+	yes	60	M	right colon	2	3	0	0	2	n.a.	neg.
29	+	yes	67	M	left colon	2	3	1	1	4	05./99	neg.
32	+	yes	57	F	right colon	3	3	1	1	4	01/02	n.d.
33	+	yes	72	M	left colon	3	3	1	1	4	03./02	neg.
36	+	yes	60	M	rectum	3	3	1	0	3	n.k./01	neg.
37	+	yes	76	F	right colon	2	3	0	0	2	06/96	neg.
38	neg.	n.d.	76	F	left colon	2	3	1	0	3	08/97	n.d.
39	+	yes	63	M	rectum	2	3	2	1	4	11/01	neg.

### Stage dependent chromosomal alterations in colorectal cancer tumours

For 19 of the initial 29 samples, the DNA quality was good enough to generate interpretable matrix-CGH-profiles. Primary tumours already showing clinical evidence of distant metastasis (UICC stage IV, n = 7) a total number of 13.6 gains or losses per tumour were detected. In contrast, the UICC stage II (n = 5) and III (n = 7) tumours only displayed an average of 10.5 chromosomal imbalances per tumour (table 2), i.e. 12.4 for stage II and 9.1 for stage III tumours, respectively. In contrast, taking only into account chromosomal regions showing gains or losses in more than 50% of all tumours we saw no significant differences in the number of stage dependent chromosomal aberrations (9.2 for stage II, 9.5 for stage III and 9.7 for stage IV tumours).

**Table 2:** Overview of the chromosomal aberrations in different tumour stages in the tumours with interpretable profiles

Stage	UICC IV	UICC II/III	Total
**Number**	7	12	19
**Gains**	70	94	164
**Losses**	25	32	57
**Total**	95	126	221
**Aberrations/tumour**	13.6	10.5	11.6

The most frequent imbalances were gains on 20q (14/19), 8q (14/19), 7p (12/19), 13q (11/19), 17q (9/19) and losses on 1p (12/19), 18q (11/19), 8p (4/19), 17p (4/19). 18q-loss (5/7 UICC IV vs. 7/12 UICC II/III) and 13q-gain (5/7 UICC IV vs. 6/12 UICC II/III) were more frequently detected in UICC IV rather than in UICC II/III tumours (these data are summarized in additional files 5 and 6).

### High-resolution mapping of the gain on 13q using interphase FISH and high-density genomic arrays

**Figure 3:**
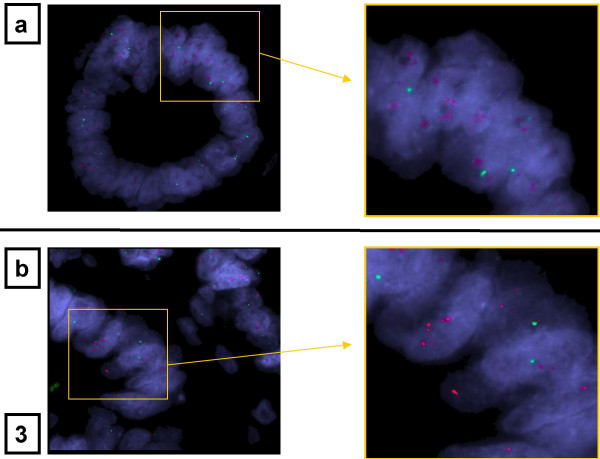
Representative figures of two-colour FISH on 2 μm-sections of a formalin-fixed tumour sample from no. 29 was performed with the Rhodamin-labeled BAC-clones RP11-89P22 (figure 3a) and RP11-8C15 (figure 3b) and compared with the FITC-labeled BAC-clone RP11-9F13 used as control in figure 3a and 3b. The Rhodamin-labeled signal appears in red and the FITC labeled signal in green (original magnification 630×). It showed the typical pattern of a high level amplification for the clones RP11-8C15 and RP11-89P22, whereas the apparently not involved clone RP11-9F13 was confirmed to be localized outside of the amplified area.

**Figure 4:**
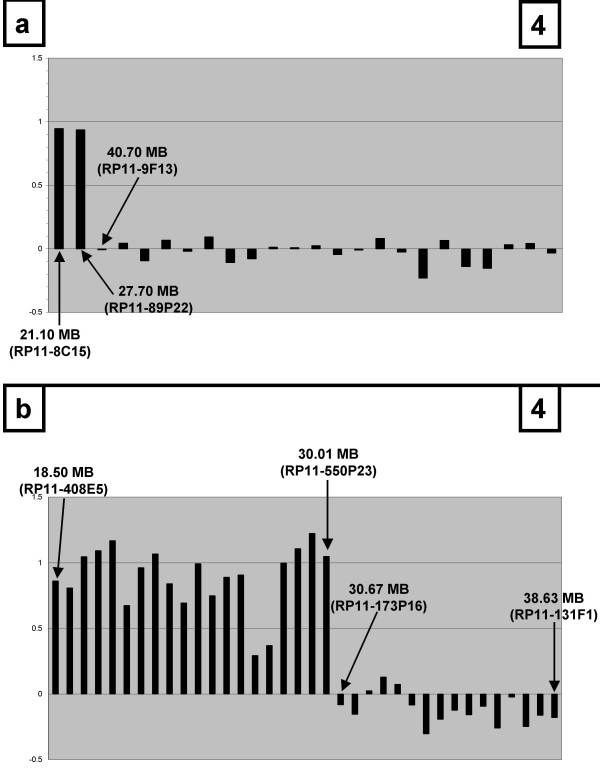
High resolution-mapping of the gain on 13q in tumour 29 using the 6 k array (figure 4b) as compared to the profile obtained with the 0.64 k genomic array (figure 4a). Log2 ratios for the different BAC clones (y-axis) are displayed according to their chromosomal position (x-axis).

In the original hybridization approach using the 644-feature array, the gain on 13q was one of the most frequent alterations in UICC IV tumours and included the large region between the BAC clones RP11-8C15 (21 Mb) and RP11-89P22 (27.7 Mb) as minimally involved area in all samples. Because the time this study was performed, this region was the least well known, we decided to confirm the validity of this array-CGH data by interphase FISH and to further characterize it. The gain in one of the tumours (no. 29) used for the initial screen with the low density arrays precisely displayed the minimally involved area from 21–27.7 Mb. The consecutive clone on this low density array located at 40.7 Mb (RP11-9F13) appeared not to be involved (Figure 4a). Interphase FISH using the 3 clones described above was done with histological sections of this particular tumour sample (Figure 3). It showed the typical pattern of a high level amplification for the clones RP11-8C15 and RP11-89P22, whereas the apparently not involved clone RP11-9F13 was confirmed to be localized outside of the amplified area.

To identify the smallest possible area of amplification and to narrow down the number of potential candidate disease genes, we repeated the matrix-CGH of this tumour sample using a higher resolution 6400-clone-chip and DNA from snap frozen tissue (Figure 4). Although it was possible to pinpoint the distal border of this gain to the area between 30.01 (BAC-clone RP11-550P23) and 30.67 Mb (RP11-173P16), the gain involved all proximal BAC clones available on this high density array up to 18.50 Mb (RP11-408E5). Thus, the aim to narrow down the minimal area harboring the disease gene was only partially achieved.

## Discussion

The presented data demonstrates that it is possible to extract DNA of sufficient quality to perform matrix-CGH from the majority of archival formalin-fixed, paraffin-embedded tumour samples collected at different institutions. This reflects the situation encountered in most multicentric therapeutic clinical trials where ample clinical and follow-up data is available, but tissue samples are either not collected systematically or at least not using a standardized protocol for tissue processing, fixation and embedding. In this study, 66% (19/29) of the tissue blocks, irrespective of the institutional origin or the age of the blocks, delivered DNA suitable for array hybridizations employing a simplified method for tumour cell enrichment involving a minimal number of tissue handling steps. The procedure of paraffin embedding and the fixation time have been shown to be able to influence the DNA quality. However, these factors could not be evaluated retrospectively. Further improvements in data quality can be realized with snap frozen samples or with paraffin-embedded tissue, once procedures for tissue processing have been standardized to ensure optimal conditions for molecular analyses and are routinely used in multicentric therapeutic trials to collect tissue. Such an approach is increasingly used by the EORTC-GI group for the PETACC-trials of adjuvant treatment for colorectal cancer patients, and among others the present study was done to verify and optimize tissue sampling protocols for genomic analyses (for example PETACC-4). Our study indicates that it should be technically feasible for the time being to use the large collections of formalin-fixed paraffin embedded tumour tissues for genomic analyses collected by the local pathologists during the routine workup of resected tumour samples of patients included in closed multicentric therapeutic trials. This possibility opens the road for systematic analyses of these archival materials with an excellent long-term follow-up to identify prognostic genomic signatures e.g. by the use of matrix-CGH immediately without the need to wait for the follow up of ongoing prospective studies.

Analysis of the array hybridizations revealed consistent regions of copy number changes. Many of the findings were in agreement with those observed previously in conventional CGH [18]and matrix-CGH [19-21]. investigations, thus confirming the validity of our hybridizations with DNA extracted from archival formalin-fixed, paraffin-embedded tumour tissue collected at multiple centers. This includes the most frequent gains observed in our study over all different tumour stages such as 20q, 8q, 7p, 13q, and 17q, as well as the most frequent losses on 1p, 18q, 8p and 17p.

Since the list of candidate genes involved in these alterations is very large and has been discussed elsewhere [19-21], we will not elaborate in a detailed discussion of individual candidates. The scientific goal of this pilot matrix-CGH study was to identify genomic signatures associated with systemically advanced colorectal tumours. Since only 15% of UICC III patients will benefit from the routinely administered systemic adjuvant chemotherapy [22], genomic signatures could serve to select patients with a high risk of recurrence who would have the major benefit from an adjuvant treatment.

Genomic profiles from primary tumours of patients with clinical evidence of metastases (UICC stage IV) differed from those of locally restricted tumours (UICC II/III) only by the type of chromosomal imbalances. The average total number of gains and losses per tumour were not different between different tumour stages, which is in agreement with a recent study of Dukes C colorectal tumours using conventional CGH. In this study, Rooney and coworkers found that the number of aberrations was highly variable. 21% of the tumours showed no aberration, whereas 41% displayed 1–8 and 38% 11–20 aberrations [23]. Interestingly, in their study patients with more than 2 aberrations appeared to have a better survival than patients with fewer regions of losses and gains.

In contrast, we found a number of imbalances that were more frequent in the UICC IV tumours. The most frequent imbalances were loss on 18q (100% UICC IV vs. 58.3% UICC II/III) and gain on 13q (85.7% UICC IV vs. 58.3% UICC II/III). Loss on 18q included the well known 18q21.1 area which is known to harbour the tumour suppressor genes *SMAD2 *[24] and *SMAD4 *[25], which function in the TGFβ-pathway e.g. to mediate the growth-inhibitory effects of this cytokine. Allelic loss at 18q21.1 and mutations of *SMAD4 *are common alterations in colorectal cancer [26]. Loss of 18q has as well been found in the recently published array-CGH analyses of colorectal cancer [19-21], though an association to more advanced stages has not yet been reported in these studies. LOH-analyses have shown that 18q allelic loss is a strong predictive factor [27]. Moreover, Dukes C tumours with SMAD4 expression show a significantly longer disease-free survival [28] and significantly more benefit from 5-fluorouracil-based chemotherapy [29].

Since at the time when we started this study little detail was available concerning the 13q gain, we decided to further characterize this alteration by high-resolution mapping using high-density genomic arrays and interphase FISH-analyses. Interphase FISH showed the typical pattern of a high-level chromosomal amplification. After finishing the experimental part of the project, a number of manuscripts were published describing genomic profiles of colorectal tumours obtained with matrix-CGH [19], all confirming the high incidence of 13q gains. However, the area described in these studies was very large and was covered by more than 20 BAC clones in most studies [20,21]. Our high resolution mapping allowed us to define the distal border of this gain to the area between 30.01 and 30.67 Mb. However, the aberration involved all proximal BAC clones available on the high density array, thus still leaving a chromosomal area of at least 11.51–11.17 Mb (between 18.50 MB and 30.01–30.67) as minimally amplified region. This area includes numerous candidate oncogenes such as *FLT3*, a tyrosine kinase receptor in which activating mutations have been found in acute myeloid leukemia [30], *FLT1*, a vascular endothelial growth factor receptor found to be expressed in gastric and breast carcinoma cells [31], and *FGF9*. FGF9 is a potent mitogen that stimulates normal and cancer cell proliferation [32] and appears to be involved in the pathogenesis of a number of tumours such as prostate cancer [33], melanomas [34], brain tumours [35], and breast cancer [36].

## Conclusion

In conclusion, the presented study shows that it is feasible to perform high-throughput analyses of genomic profiles of colorectal tumours using archival formalin-fixed, paraffin-embedded tissue samples, such as those collected by institutional pathologists from patients participating in multicentric clinical trials. The results obtained for the gain on 13q show that relevant data can be obtained using this setting, which is as well confirmed by the fact that the results obtained in this study are comparable to published genomic profiles obtained with ideal material such as snap frozen tissue. The amplification on 13q appears to harbour candidate genes that may confer a more aggressive phenotype to colorectal cancer cells. Further studies with larger series of patients are warranted to identify the relevant oncogene.

## Competing interests

The author(s) declare that they have no competing interests.

## Authors' contributions

HF was involved in the design of the study, carried out the array hybridizations, data Acquisition and Evaluation, the FISH-experiments, the MSI-testing and drafted the manuscript.

BR was responsible for the array production.

JS performed the macrodissection of the tumour specimen.

MB was involved in the FISH experiments and in drafting the manuscript.

DEA, CJ, BN and CB carried out tumour sampling and provided information about the study samples.

FR provided tumor samples, performed the DNA-extraction of the fresh frozen tissue and was involved in the design of the study.

EVC participated in the design of the study.

CHK was involved in tumor sampling and participated in the design of the study.

HAK was involved in data acquisition and evaluation.

CS was involved in the array production.

MN participated in data acquisition and evaluation.

MPL participated in the design of the study and drafting the manuscript.

PL participated in the design of the study, in array production and evaluation.

TMG conceived the study and participated in its design and coordination and in drafting the manuscript.

All authors read and approved the final manuscript.

## Pre-publication history

The pre-publication history for this paper can be accessed here:

http://www.biomedcentral.com/1471-2407/7/58/prepub

## Supplementary Material

Additional file 1 DNA extraction protocol. Detailed description of the protocol for DNA extraction in paraffin embedded tissue used in this study. Click here for file

Additional file 2 Clone list standard chip. List of the clones included in the standard chip together with their genomic localization. Click here for file

Additional file 3 Clone list high resolution chip. List of the clones included in the high resolution chip together with their genomic localization. Click here for file

Additional file 4 Array hybridization protocol. Detailed description of the protocols for array hybridization. Click here for file

Additional file 5 Table 3. Genomic imbalances (1p to 22q) found by matrix-CGH of 19 cases of colon cancer, summarizing the chromosomal regions affected by loss and gain in more than one third of the patients. The table is a subset of additional file 6 (table 4). The cloned sequences are aligned in chromosomal order from 1p to 22q. They are denoted by their clone ID (RP) and their chromosomal localization. Their measured state of imbalance for patient 1 to 19 is displayed by color coding: white, balanced; orange, lost; green, gained. Black boxes, test sequences missing on the chip; gray boxes, sequences not evaluated due to poor quality. Click here for file

Additional file 6 Table 4. Genomic imbalances (1p to 22q) found by matrix-CGH of 19 cases of colon cancer. The cloned sequences are aligned in chromosomal order from 1p to 22q. They are denoted by their clone ID (RP) and their chromosomal localization. Their measured state of imbalance is displayed by color coding: white, balanced; orange, lost; green, gained. Black boxes, test sequences missing on the chip; gray boxes, sequences not evaluated due to poor quality. Click here for file
