# Supplementary material for: Matrix-comparative genomic hybridization from multicenter formalin-fixed paraffin-embedded colorectal cancer tissue blocks
Source: BMC Cancer. 2007 Apr 2;7:58. doi: 10.1186/1471-2407-7-58 (PMC3225877; doi:10.1186/1471-2407-7-58)
Supplement: Additional file 1 — DNA extraction protocol. Detailed description of the protocol for DNA extraction in paraffin embedded tissue used in this study. [file 1471-2407-7-58-S1.doc]

# DNA Extraction from paraffin embedded tissue

Paraffin was removed from the sections by the following procedure: Sections were transferred to Eppendorf tubes and washed with 1ml xylol (10 minutes, 2 times) and 1 ml ethanol (5 minutes, 2 times). After each washing step, tissue material was recovered by centrifugation (5 min., 13000 rpm). After the last washing step, the material was dried and incubated in 1 ml NaSCN (1M) for 12 hours.

After removing the NaSCN and washing 2 times with 1 ml DNA isolation buffer, the sections were suspended in DNA isolation buffer containing 30 µl proteinase K solution (20 mg/ml) for 72 hours at 55°C. 10 µl aliquots of proteinase K solution were repeatedly added after 24 and 48 hours.

DNA purification was performed by Phenol extraction. The solution was distributed into two tubes and washed with 1 ml phenol (pH = 7.5) (20-30 minutes, 2 times), 500 µl phenol + 500 µl chlorophorm/ isoamylacohol (24:1) (20-30 minutes, once) and 1 ml chloroform (20-30 minutes, 2 times). After each washing step, the aquaeous phase was carefully transferred to a fresh tube. Finally, DNA was recovered by ethanol precipitation and resuspended in 50 µl TE-buffer.
