# Supplementary material for: Matrix-comparative genomic hybridization from multicenter formalin-fixed paraffin-embedded colorectal cancer tissue blocks
Source: BMC Cancer. 2007 Apr 2;7:58. doi: 10.1186/1471-2407-7-58 (PMC3225877; doi:10.1186/1471-2407-7-58)
Supplement: Additional file 4 — Array hybridization protocol. Detailed description of the protocols for array hybridization. [file 1471-2407-7-58-S4.doc]

# Array hybridization protocol

Tumor DNA or cell-line DNA and reference DNA (opposite sex-matched) from healthy donors were differentially labeled with Cy3-/Cy5-conjugated dCTP by use of a BioPrime DNA Labeling Kit (Invitrogen, Karlsruhe, Germany). Unincorporated nucleotides, random primers, and dyes were removed by use of MicroCon YM30 Spin Columns (Millipore, Schwalbach, Germany). Incorporation of dyes into labeled DNA was controlled by UV spectrophotometry.

Tumor and reference DNA were combined with 75 µg of human Cot-1 DNA (Roche Diagnostics, Mannheim, Germany) and suspended in 120 µl of Ultrahyb™ hybridization buffer (Ambion, Bad Soden, Germany). Denaturation of combined DNA was performed at 75° C for 10 minutes, followed by preannealing at 37° C for 60 minutes. Hybridization was carried out in a GeneTAC Hybridization Station (Genomic Solutions, Oberhaching, Germany) for 40-48 hrs at 37° C.

Subsequently, slides were washed for four cycles in 2x SSC, 50% formamide, and 0.1% Tween 20 (pH 7.0): The first cycle at 37° using a flow time of 30 s and a hold time of 1 s, the second cycle at 45°C using a flow time of 1 s and a hold time of 3 min and the next two cycles at 45°C using a flow time of 20 s and a hold time of 3 min. Finally, slides were washed at 25° C in 1x PBS, 0.05% Tween 20 (pH 7.0) using a flow time of 2 min. and a hold time of 1 s. Slides were dried in a centrifuge by spinning for 5 min at 2000 x g.
